# Determinants of Anemia among Pregnant Women Attending Antenatal Clinic in Public Health Facilities at Durame Town: Unmatched Case Control Study

**DOI:** 10.1155/2018/8938307

**Published:** 2018-09-24

**Authors:** Fekede Weldekidan, Mesfin Kote, Meseret Girma, Negussie Boti, Teklemariam Gultie

**Affiliations:** ^1^Department of Public Health, College of Medicine and Health Science, Arba Minch University, Arba Minch, Ethiopia; ^2^Department of Midwifery, College of Medicine and Health Sciences, Arba Minch University, Arba Minch, Ethiopia

## Abstract

**Background:**

Anemia among pregnant women is one of the most common public health problems in developing country. World health organization (WHO) estimate shows nearly half of pregnant women were affected by anemia. High burden of anemia is observed in Africa particularly in Ethiopia. However, the factors that contribute to the occurrence of anemia were not exhaustively studied. Therefore, the aim of this study was to identify determinant factors of anemia among pregnant women in Durame Town, southern Ethiopia.

**Method:**

An institutional based unmatched case control study was conducted among 111 cases and 222 controls in Durame Town from 16th February to 8th May 2017 using interviewer administered questionnaires. Controls were pregnant women whose hemoglobin level was 11 g/dl and above at their first antenatal care clinics and cases were pregnant women whose hemoglobin level was less than 11 g/dl. Bivariate and multivariate logistic regression model was used to see the determinants of anemia. Adjusted odds ratio (AOR) with 95% confidence interval (CI) and p-value were used to identify the significant association.

**Result:**

A total of 333 women (111 cases and 222 controls) participated in the study. The major determinant factors were parasitic infection (AOR: 3.67, 95% CI: 1.72-7.86), not taking additional diet during pregnancy (AOR: 2.49, 95% CI: 1.22-5.08), consuming tea/coffee immediately after food (AOR: 3.58, 95% CI: 1.72-7.42), not eating meat (AOR: 2.07, 95% CI: 1.03-4.15), previous heavy menstrual blood flow (AOR: 2.62, 95% CI: 1.18-5.84), and being housewife (AOR: 2.38, 95% CI: 1.02-5.57).

**Conclusion:**

Parasitic infection, additional diet during pregnancy, consuming tea/coffee immediately after food, meat consumption, previous heavy menstrual blood flow, and occupational status of women were significant factors associated with anemia among pregnant women. Therefore, anemia prevention strategy should include promotion of counseling on additional diet during pregnancy, preventing parasitic infection, and increasing employment opportunities for women.

## 1. Introduction

Anemia in pregnancy is one of the most common preventable causes of maternal morbidity and poor prenatal outcome [1]. Anemia is defined as a decrease in the concentration of circulating red blood cells or in the hemoglobin concentration and a concomitant impaired capacity to transport oxygen because of low level of circulating erythrocyte than the normal [1, 2]. World Health Organization has accepted up to 11 gm percent as the normal hemoglobin level in pregnancy. Therefore any hemoglobin level below 11 gm in pregnancy should be considered as anemia [3].

Anemia is a global public health problem affecting both developing and developed countries with major consequences for human health as well as economic development [2]. According to WHO report about 56 million pregnant women are affected by anemia worldwide. Among those 17.2 million pregnant mothers are from Africa [4].

Anemia in pregnancy is a condition with effects that may be deleterious to mothers and fetuses and it is a known risk factor for many maternal and fetal complications [2]. It significantly increases fetal and maternal mortality and morbidity due to maternal vulnerability for infection and hemorrhage [5]. The nutritional status of women in Ethiopia is low and their daily workload is often enormous because of reproducing and ensuring the survival of their children. Due to these facts, in Ethiopia, iron deficiency anemia is the commonest problem affecting pregnant women, women of reproductive age, and children [6–8]. The Federal Ministry of Health develops a national nutrition program strategy to improve the micronutrient deficiency among pregnant women by increasing the system to give comprehensive and routine nutritional assessment intervention as well as routine iron and folic acid supplementation and deworming during pregnancy [9].

Despite the efforts made by the government and other stakeholders, anemia during pregnancy is still a public health problem in Ethiopia [9]. According to the Ethiopian Demographic and Health Survey (EDHS) report, about one-fourth (23%) of reproductive age women are anemic [10]. This is mainly due to poor nutritional intake, repeated infections, menstrual blood loss, and frequent pregnancies. It is also associated with socioeconomicconditions and health related factors during their pregnancy [11–13]. However, the availability of local information on the determinant factors still no adequate to prevent Anemia and its consequence. Therefore, the aim of this study was to identify determine factors of Anemia among pregnant women attending antenatal care in public health care facilities of Durame Town, southern Ethiopia.

## 2. Methods

### 2.1. Study Area and Design

This study was conducted in public health facilities of Durame Town, Kembata Tembaro zone, southern Ethiopia, from February 16 to May 08, 2017. Durame Town is the capital city of Kembata Tembaro Zone. In Durame there are three health centers, one hospital, and four private clinics. The catchment area of the hospital is Kembata Tembaro Zone and that of the three health centers is Durame Town. Durame General Hospital is the only public hospital serving as referral hospital for Kembata Tembaro Zone. Durame Town is located 309 km south of Addis Ababa and 126 km far from Hawassa city which is the capital city of SNNPR. The altitude of the area is 2213 m above sea level. According to report from CSA, the total population of the town is about 46638 people [14]. According to zone health department report, 1596 women in the town are expected to be pregnant in the study period. The four health institutions are the only institutions which provide ANC service for pregnant women in Durame Town. An institutional based case control study was used to identify the determinants of anemia among women attending their first ANC.

### 2.2. Source and Study Populations

All pregnant women aged 15–49 years who were receiving their first antenatal care in Durame town were the source population. The study population was pregnant women aged 15–49 years who were receiving their first antenatal care in Durame town during February16 to May 08, 2017. Pregnant women who were severely ill and those with 2nd and 3rd visits were excluded from the study.

### 2.3. Sample Size Determination

The sample size was calculated using the double population proportion approach and it was calculated using Epi Info version 7.02 statistical software package with the assumption of 95% confidence level (Z*α*/2= 1.96), 80% power (Z*β*= 0.84), case to control ratio of 1:2 (r = 2), the odds ratio = 2 from factors that has association with anemia from recent study conducted in Butajira Ethiopia and proportion of cases with exposure 43.5% [15]. Considering 10% nonresponse rate, the maximum sample size was 111 cases and 222 controls with a total of 333 study participants.

### 2.4. Sampling Procedure

Systematic random sampling technique is used to select study participants using their average previous month's antenatal care users used as the sampling frame. In Durame Town there were four public health facilities that give antenatal care service to the pregnant mother. All health facilities found in the town were included and sample was allocated proportionally to population across four health facilities. Cases were selected using systematic random sampling technique and two consecutive controls who came after selection of cases were allocated for each cases.

### 2.5. Operational Definition

Anemia was defined according to WHO criteria [16]. Using WHO criteria, pregnant women whose hemoglobin level was 11 g/dl and above at their first antenatal care clinics were selected as control (non-anemic) and those with a hemoglobin level less than 11 g/dl were selected as case (anemic).

### 2.6. Data Collection Procedure and Data Quality Control

A structured pretested interviewer administered questionnaire was developed in English and then translated into Amharic language for simplicity then back-translated to English language for its consistency by two different language expert individuals who speak both English and Amharic fluently. The questionnaire has four parts sociodemographic, obstetrics characteristics, nutritional related characteristics, and parasitic infection related characteristics were used. Pretesting of the questionnaire was done on 5% of the sample among ANC attendees who attend ANC at Kedida Gamella district who were not included in the study; that was a week before commencement of the actual data collection. Based on the pretest, a questionnaire was corrected to ensure clarity, wording, and logic sequence and skip patterns. Data was collected using exit interview by 10 diploma midwives and supervised by two trained health professionals who had BSc. All data collectors & supervisors were trained for one day and performed practical exercises to be familiar with the questionnaire. The data collectors were regularly supervised for proper data collection; all the questionnaires were checked for completeness and consistency in daily basis.

### 2.7. Specimen Collection and Processing

The specimen collection and processing were carried out by two trained laboratory technologists. Each step of specimen collection, processing, and analysis was supervised by experienced and trained laboratory technologist. The blood for hemoglobin concentration was done based on the Standard Operational Procedures (SOPs). A venous blood sample was taken from the study participants; using a HemoCue Hb 301 analyzer (manufactured by HemoCue AB), a precalibrated instrument was designed for the measurement of hemoglobin concentration and labeled with identification number. Venous blood was drawn, through microcuvettes, and inserted into the HemoCue Hb analyzer and the result was recorded. Stool samples were collected by using a clean and labeled container from the study participants. A portion of the stool was processed with direct microscopic technique to detect intestinal parasites immediately. For detection of helminthes, eggs, larvae, and cysts of protozoan parasites, the samples were examined microscopically first with 10x and then with 40x objective. The remaining part of the sample was emulsified in a 10% formalin solution. Stool examinations were done using formal ether concentration technique, which is considered the most sensitive for most intestinal helminthes. The same method was carried out across all centers.

### 2.8. Data Processing and Analysis

The collected data were coded, cleaned, and entered by Epi-Info version 7.2 and exported to statistical package for social science (SPSS) version 20.0 for analysis. Descriptive statistics including graphs, charts, tables, and proportion was used to describe the data. Bivariate and multivariate logistic regression analysis was performed to see the association between dependent and independent variables. Variables that showed association in bivariate analysis and which have P-value less than 0.20 were entered into multivariate logistic regression model [17]. Finally multivariate logistic regression model is used for controlling confounding factors and to identify significant factors associated with Anemia. An effort was made to assess whether the necessary assumptions for the application of multivariate logistic regression were fulfilled. In this regard, the Hosmer and Lemeshow's goodness-of-fit test with large p-value (p>0.05) was checked to see good fitness. Multicollinearity and confounding effect was checked by using Standard error. The variable without multicollinearity was entered into multivariate model. At the end AOR with 95% CI, P-value <0.05 was considered statistically significance.

### 2.9. Ethical Consideration

The study was conducted after getting ethical clearance from Arba Minch University, College of Medicine and Health Science Institutional Review Board (IRB). Support letter was obtained from Zonal Health Department as per the recommendation letter from the public health department. Written informed consent was secured from study participants after explaining about the objective and purpose of the study to each study participant. The participants were also assured about the confidentiality of the data. While assessing status of anemia, the result of the test was communicated immediately to each participant and if the pregnant woman was anemic as well as infected with parasites, they were referred to the health personnel for treatment and follow-up.

## 3. Result

### 3.1. Sociodemographic Characteristics of the Study Participants

A total of 333 respondents (111 cases and 222 controls) participated in the study with 100% response rate. Seventy-six (68.8%) cases and 176 (71.6%) controls were in the age group between 20 and 29 years. The dominant ethnic group of the participant were Kembata in both groups, 92 (82.9%) cases and 175 (78.8%) controls. With regard to religion, 101 (91%) cases and 176 (79.3%) controls were Protestants. Pertaining to maternal educational status, 40 (36%) cases and 107 (48.2%) controls were at the primary educational level. Regarding educational status of husband, 44 (39.6%) cases and 108 (48.6%) controls have completed primary educational level. Concerning occupational status of the participant, 35 (31.5%) cases and 89 (40.1%) controls were housewives (Table 1).

### 3.2. Obstetrics Related Characteristics of the Study Participants

Among pregnant mothers who were receiving antenatal care at health facilities, 86 (77.5%) cases and 156 (70.9%) controls had previous history of pregnancy. From those who had history of birth, nearly one-third of the cases, 33(38.4%), and 125 (80.1%) controls had birth interval of more than two years. Among those who had history of birth, 53 (47.7%) cases and 155 (69.8%) controls had the less than or equal to four children. Among ANC attendees, 43 (38.7%) cases and 125 (56.3%) controls had history contraceptive use. More than half of the ANC attendees, 73 (65.8%) cases and 195 (87.8%) women, had no heavy menstrual flow before they become pregnant. Concerning gestational age, the majority of the participants, 50 (45%) cases and 93 (42%) controls, were at second trimester of their first antenatal care booking (Table 2).

### 3.3. Nutritional Related Characteristics of the Study Participants

Majority, 88 (79.3%), of cases and 20 (9%) controls were not consuming additional food during current pregnancy. Majority of pregnant women who received ANC, 72 (64.9%) cases and 77 (34.7%) controls, had not eaten meat at least one day per week. Regarding the tea/coffee consumption, 83 (74.8%) cases and 110 (49.5%) controls were taking tea/coffee immediately after food every day (Table 3).

### 3.4. Parasitic Infection Related Characteristics of the Study Participants

Forty-nine (44.1%) cases and 35 (15.8%) controls had intestinal parasitic infection. Among intestinal parasites detected in the pregnant women, 19 (17.1%) cases and 17 (7.7%) controls were infected by* Ascaris lumbricoides* and 14 (12.6%) cases and 10 (4.5%) controls were infected by G*iardia lamblia*. More than quarter of the attendees, 37 (33.3%) cases and 39 (17.6%) controls, had history of malaria attack in the last two months (Table 4).

### 3.5. Determinants of Anemia during Pregnancy

Bivariable logistics regression was performed for each independent variable. Variables having a p-value of less than 0.25 were transferred to multivariable logistic regression. The results of multivariable logistic regression with backward method after checking of model fitness test by Hosmer and Lemeshow as well as omnibus test which had nonsignificant and significant test result were tested, respectively. And also multicollinearity was checked by using standard error of greater than two indicating that there was no multicollinearity.

The result in multivariable logistic regression showed that the odds of getting anemia in pregnant mothers were higher among mothers whose birth interval was less than two years (AOR=4.78, 95% CI:2.68 -9.64). This study showed that pregnant women who had heavy menstrual blood flow were 2.6 times more likely to have a risk of developing anemia than who did not have history of heavy menstrual blood flow (AOR=2.62, 95% CI:1.18 – 5.84).

According to this study odds of getting anemia in pregnant mothers who did not consume additional food during their pregnancy were 2.5 times greater than the odds of mothers who consume additional food at least one times per day during their pregnancy (AOR=2.49,95% CI:1.22- 5.08). Similarly, pregnant mothers who were not eating meat at least one time per week had two times more likelihood to develop anemia than who ate meat at least one times per week (AOR=2.07, 95% CI: 1.03 – 4.15). The odds of developing anemia among pregnant mothers who were consuming tea/coffee immediately after food were 3.6 times greater than the odds of mothers who did not consume tea/coffee immediately after food during their current pregnancy (AOR=3.57, 95% CI: 1.72 – 7.42).

In this study parasitic infection was also found to be a significant determinant of anemia. Those mothers who were infested by parasitic infection were 3.7 times more likely to develop anemia than those who were not infested by parasitic infection during the current pregnancy (AOR=3.67, 95% CI: 1.72- 7.85) (Table 5).

## 4. Discussion

In order to effectively prevent anemia during pregnancy, its important to identify the factors which contribute to the development of anemia. In this study, pregnant women who had heavy menstrual blood flow before the index pregnancy were 2.6 times more likely to have a risk of developing anemia than who did not have history of heavy menstrual blood flow. This finding is consistent with previous studies conducted in southern and southeast Ethiopia which showed that having heavy menstrual flow was more likely to develop anemia [15, 18–20]. The possible reason may be that heavy menstrual blood flow leads a woman to heavy blood loss, which in turn leads to anemia.

In this study, birth interval was found to be a significant determinant of anemia. Those pregnant mother whose birth interval was less than two years were five times more likely to develop anemia than whose birth interval reported to be greater than two years. This finding is consistent with previous studies conducted in Addis Ababa and Sudan [7, 8, 21]. This is due to the fact that short intervals between births may not provide women with enough time to replenish lost nutrient stores before another reproductive cycle begins

According to this study, odds of getting anemia in pregnant mothers who did not consume additional food during their pregnancy were 2.5 times greater than the odds of mothers who consume additional food at least one times per day during their pregnancy. This result is consistent with previous studies conducted in Mekele town and study conducted in Hawassa and Yirgalem cities of southern Ethiopia [11, 12]. This might be due to the fact that pregnancy is a special period with increased energy and nutrient requirement which can be fulfilled with increased meal frequency

From this study, it was observed that pregnant housewives were two times more likely to develop anemia than who have job. The same result was documented in retrospective case control study done in Turkey and Mekelle town in Ethiopia [7, 22, 23]. The possible reason may be that housewives may have financial constraint, work load and may not have early access to health care services.

Pregnant women who were not eating meat at least one time per week compared to those who ate two times had the likelihood of developing anemia. This result is consistent with other studies done in Ethiopia [12, 13, 24]. This significant association might be due to the reason that meat is an important source of heme iron.

However, consuming tea/coffee immediately after food has a negative association with anemia during pregnancy. The odds of developing anemia among pregnant mothers who were consuming tea/coffee immediately after food were 3.6 times greater than the odds of mothers who did not consume tea/coffee immediately after food during their current pregnancy. This result is in agreement with a study done in Egypt and Ethiopia, which showed significant association between anemia and consumption of tea [19, 25]. This could be drinking tea/coffee after food intake may affect iron absorption which leads to inadequate dietary iron intake in the pregnant women.

There was a strong significant association between intestinal parasitic infection and anemia in pregnant women. Those mothers who were infected by parasitic infection were 3.7 times more likely to develop anemia than those who were not infected by parasitic infection during the current pregnancy. This finding is in agreement with a study done in Yirgalem and Hawassa cities, Dessie town and Canada [12, 23, 26]. This might be due to blood loss caused by parasitic infestations that might put mothers at high risk of iron deficiency anemia.

## 5. Conclusion

This study identified important factors that determine anemia among pregnant women in the study area. Among the studied factors, drinking tea/coffee immediately after food intake, not eating meat, not taking additional diet during current pregnancy, presence of parasitic infestations, short birth interval, previous heavy menstrual blood flow, and being housewife are the factors which was associated with anemia during pregnancy. It is recommended that community based health education and mass deworming of intestinal parasites is important for prevention of anemia in pregnancy. A community based study with large sample size and more strong study design should be conducted.

## Figures and Tables

**Table 1:** Socio-demographic characteristics of pregnant mother attending ANC in public health facilities of Durame Town, February to May 2017.

**Variable**	**Categories**	**Cases (n=111)**	**Controls (n=222)**
		**N= (**%**)**	**N= (**%**)**
**Age **	15 – 19	8 (7.2)	9 (4.1)
	20 – 24	41 (37)	67 (30.2)
	25 – 29	35 (31.5)	92 (41.4)
	30 – 35	23 (20.7)	44 (19.8)
	>35	4 (3.6)	10 (4.5)

**Residence **	Urban	69 (62.2)	138 (62.2)
	Rural	42 (37.8)	84 (37.8)

**Ethnicity**	Kembata	92 (82.9)	175 (78.8)
	Tembaro	5 (4.5)	21 (9.5)
	Hadiya	9 (8.1)	25 (11.3)
	Other*∗*	5 (4.5)	1 (10.5)

**Religion **	Protestant	101 (91)	176 (79.3)
	Catholic	6 (5.4)	23 (10.4)
	Orthodox	2 (1.8)	15 (6.8)
	Others*∗∗*	2 (1.8)	8 (3.6)

**Maternal educational level**	Have no formal education	37 (33.3)	45 (20.3)
	Primary	40 (36.0)	107 (48.2)
	Secondary and above	34 (30.6)	70 (310.5)

**Husband education level**	Have no formal education	32 (28.8)	25 (11.3)
	Primary	44 (39.6)	108 (48.6)
	Secondary and above	35 (31.6)	89 (40.1)

**Occupational status **	House wife	35 (31.5)	89 (40.1)
	Merchant	25 (22.5)	80 (36)
	Employed	38 (34.2)	45 (20.3)
	Other*∗∗∗*	13 (11.7)	8 (3.6)

*∗*others, Amhara, Silt, Halba, sidama, *∗∗*Adventist, Muslim, *∗∗∗*Farmer, Day laborer.

**Table 2:** Obstetrics related characteristics of pregnant mother attending ANC in public health facilities of Durame Town, February to May 2017.

**Variable**	**Categories**	**Cases (n=111)**	**Controls (n=222)**
		**Frequency (**%**)**	**Frequency (**%**)**
**History of previous pregnancy**	Yes	86 (77.5)	156 (70.9)
	No	25 (22.5)	66 (29.7)

**Birth interval between the last and current**	Primigravida	25 (22.5)	66 (29.7)
	< 2 year	53 (47.7)	31 (14)
	>2 year	33 (29.7)	125 (56.3)

**Number of children**	≤ 4	53 (47.7)	155 (69.8)
	>4	58 (52.3)	67 (30.2)

**Contraceptive use**	Yes	43 (38.7)	125 (56.3)
	No	68 (61.3)	97 (43.7)

**Previous heavy menstrual flow **	Yes	38 (34.2)	27 (12.2)
	No	73 (65.8)	195 (87.8)

**Gestational age**	1^st^ trimester	14 (12.6)	44 (19.8)
	2^nd^ trimester	50 (45 )	93 (42)
	3^rd^ trimester	47 (42.4)	85 (38.2)

**Table 3:** Nutritional related characteristics of pregnant mother attending ANC in public health facilities of Durame Town, February to May 2017.

**Variable**	**Categories**	**Cases (n=111)**	**Controls (n=222) **
		**Frequency (**%**)**	**Frequency (**%**)**
**Additional food during pregnancy**	Yes	23 (20.7)	202 (91)
	No	88(79.3)	20 (9)

**Meat consumption per week **	Don't eat	72 (64.9)	77 (34.7)
	Eat one days per week	39 (35.1)	145 (65.3)

**Tea/coffee consumption immediately after food **	Don't drink	28 (25.2)	112 (50.5)
	Drink	83 (74.8)	110 (49.5)

**Table 4:** Parasitic infection related characteristics of pregnant mother attending ANC in public health facilities of Durame Town, February to May 2017.

**Variable**	**Categories **	**Cases (n=111) **	**Controls (n=222)**
		**N (**%**)**	**N (**%**)**
**Intestinal parasite on current pregnancy**	Yes	49 (44.1)	43 (19.4)
	No	62 (55.9)	179 (80.6)

**Stool examination**	Ascaris Lumbricoides	19 (17.1)	17 (7.7)
	Hook worm	11 (9.9)	5 (2.3)
	Giardia Lamblia	14 (12.6)	10 (4.5)
	Mixed infection	5 (4.5)	11 (5.0)
	No parasite	62 (55.9)	179 (80.6)

**Malaria attack in the last 2 months**	Yes	37 (33.3)	39 (17.6)
	No	74 (66.7)	183 (82.4)

**Table 5:** Determinants of anemia among pregnant mothers attending ANC in public health facilities of Durame Town, February to May 2017.

**Variable**	**Cases **	**Controls**	**COR (95**%**CI)**	**AOR (95 **%** CI)**	**P – value **
**Birth interval**					

≤ 2 year	53 (47.7)	31 (14)	6.4 (3.6-11.6)	4.77 (2.68-9.64)*∗*	0.01

>2 year	33 (29.7)	125 (56.3)	1	1	

**Previous heavy menstrual flow **					

Yes	38 (34.2)	27 (12.2)	3.76 (2.14 -6.6)	2.62 (1.18,5.84)*∗*	0.01

No	73 (65.8)	195 (87.8)	1	1	

**Occupational status of women **					

House wife	94 (84.7)	130 (58.6)	3.9 (2.2-6.99)	2.5 (1.01– 5.56)*∗*	0.04

Has work	17 (15.3)	92 (41.4)	1	1	

**Additional food during pregnancy**					

Yes	23 (20.7)	202 (91)	1	1	

No	88 (79.3)	20 (9)	2.88 (1.8 - 4.6)	2.5 (1.22-5.08 )*∗*	0.01

**Meat consumption per week**					

Don't eat	72 (64.9)	77 (34.7)	4.0 (2.47-6.59)	2.1 (1.1 – 4.15)*∗*	0.04

Eat one days per week	39 (35.1)	145 (65.3)	1	1	

**Tea/coffee consumption immediately after food**					

Don't drink	28 (25.2)	112 (50.5)	1	1	

Drink	83 (74.8)	110 (49.5)	3.02 (1.83 - 5)	3.6 (1.72 - 7.41)*∗*	0.01

**Parasitic infection**					

Yes	49 (44.1)	35 (15.8)	4.223 (2.5-7.1)	3.7 (1.7- 7.85)*∗*	0.01

No	62 (55.9)	187 (84.2)	1	1	

AOR = adjusted odd ratio; CI = confidence interval; COR = crude odd ratio; *∗* = statistically significant.

## Data Availability

The data used to support the findings of this study are available from the corresponding author upon request.

## Abbreviations

ANC: Antenatal care
AOR: Adjusted odds ratio
CI: Confidence interval
COR: Crude odds ratio
EDHS: Ethiopia Demographic and Health Survey
OR: Odds ratio
WHO: World Health Organization.
